# UK autumn statement 2022, the worst is yet to come

**DOI:** 10.1177/17579139231157525

**Published:** 2023-03-31

**Authors:** R Machin

**Affiliations:** Department of Social Work, Care and Community, Nottingham Trent University, 50 Shakespeare Street, Nottingham NG1 4FQ, UK. Email: richard.machin@ntu.ac.uk

*In the following article, Richard Machin considers the public health implications of the Autumn Statement, issued by the new UK government on 17 November 2022*.

The Autumn Statement of 17 November 2022 affords us the first significant opportunity to judge the impact of Prime Minister Rishi Sunak’s policies on population health and inequality. The public health challenges facing the Government are immense. Even within the last few months there have been reports linking 330,000 excess deaths to austerity policies,^1^ the issuing of a record numbers of emergency food parcels,^2^ unprecedented rates of in-work poverty,^3^ and a coroner’s verdict that the tragic death of Awaab Ishak was caused by a respiratory condition due to the effects of mould in his Rochdale home.

The International Monetary Fund recognises that inequality should be tackled from the bottom up with growth resulting from increasing the income share of the poor.^4^ A decade of funding cuts from 2010 onwards resulted in reduced life expectancy and increased health inequalities.^5^ A second round of austerity will inevitably bring a deterioration in the social determinants of health (including child poverty, fuel poverty, unaffordable housing, and poor working conditions).

Any positives of the Autumn Statement need to be put in the context of recent history and expected future political choices. The stark facts are that the Autumn Statement introduced tax rises and spending cuts of around £50 billion; household disposable income is expected to fall by seven percent over the next two years, and living standards this year are expected to be the worse since records began.^6^ Welfare benefits will rise with inflation (although not until April 2023), but this is only the fourth such rise in 10 years and their value is six percent lower than before the COVID-19 pandemic.^7^ The additional £3.3 billion funding for the National Health Services (NHS) is welcome but is set against a two year delay in the cap on social care costs and a long-term shortfall in funding. UK health spending in the decade before the pandemic was 18% below the European Union (EU) average.^8^ In real terms, on a per person basis, the public health grant has been cut by 25% since 2015/2016, with the most deprived areas of the UK typically experiencing the most significant cuts.^9^ As of, 1 December, the energy price cap is extended for one year but on a less generous basis, meaning household energy bills will increase by an average of 20%.

Despite the current economic turmoil and change of Prime Minister, an ambitious agenda for public health must be a priority for the UK government. However, the Autumn Statement made no explicit mention of public health and the reported shelving of the white paper on health inequalities further demonstrates a lack of commitment to addressing population health. Population health relies on the government appropriately responding to the cost-of-living-emergency; improvements in health will only come with the right policy decisions, and properly funded public health and social care sectors. Public health scrutiny of government policy is more important than ever and will remain the case as most of the spending cuts announced in the Autumn Statement are scheduled for April 2025.^10^
